# Improvements in vertical jump by healthy adults at high altitude despite inter‐individual differences in neuromuscular coordination changes

**DOI:** 10.14814/phy2.70866

**Published:** 2026-04-16

**Authors:** Michele D'Attilio, Riccardo Rua, Alberto Taverna, Vittore Verratti, Danilo Bondi

**Affiliations:** ^1^ Department of Innovative Technologies in Medicine and Dentistry University “G. d'Annunzio” Chieti–Pescara Chieti Italy; ^2^ Medical Specialty School of Anaesthesia, Resuscitation, Intensive Care and Pain Management University of Torino Torino Italy; ^3^ Medical Specialty School of Anaesthesia, Resuscitation, Intensive Care and Pain Management University of Trento Trento Italy; ^4^ Department of Science University “G. d'Annunzio” Chieti–Pescara Chieti Italy; ^5^ Italian Society of Mountain Medicine Padova Italy; ^6^ Center for Disability, Rehabilitation, and Sports Medicine (CARES) University “G. D'Annunzio” Chieti–Pescara Chieti Italy; ^7^ Department of Neurosciences, Imaging and Clinical Sciences University “G. d'Annunzio” Chieti–Pescara Chieti Italy; ^8^ Department of Life, Health and Environmental Sciences University of L'Aquila L'Aquila Italy

**Keywords:** CMJ, gait analysis, hypobaric hypoxia, standing balance, strength symmetry

## Abstract

Research indicates that the performance is enhanced in situations involving rapid movements at high altitude. However, maintaining equilibrium and walking economically in low‐oxygen conditions are often impaired. The objective of this study was to evaluate the impact of trekking at high altitudes on static balance, vertical jump, gait, and maximal strength symmetry by using portable technologies. Fifteen healthy travelers (eight men and seven women, aged 41.67 ± 14.71 years) completed a high‐altitude trekking expedition up to 5000 m and were tested for one‐leg standing balance, gait, repeated counter‐movement jumps, and symmetry of maximum plantar flexion strength of the ankle at peak altitude (HA) and at low altitude (before and after the high‐altitude trek, LApre and LApost). The strategy developed for balance maintenance was focused on sagittal tilt. Gait analysis revealed an increase in hip range of motion in the sagittal and transverse planes at HA. Jump height increased from LApre to HA and LApost, while maximal strength symmetry exhibited non‐homogeneous inter‐individual changes. The observed increments in vertical jump were attributed to the beneficial effect of trekking, rather than to reduced air density. The use of portable instruments represents a significant advancement for research into motor control and physical performance at high altitude.

## 1 INTRODUCTION

High altitude poses challenges to athletic performance which affect endurance performance more than power or strength activities. However, improved performances in rapid actions as jumps have been reported, possibly due to reduction in external resistance to movement and to the modified muscle recruitment pattern, which in turn can rely on air density decrement and increased anaerobic metabolism (Feriche et al., 2014). Enhanced performance in vertical jump performance at an altitude of 2320 m has been attributed to increased maximal power of the leg muscles through augmented capabilities to generate velocity rather than force (García‐Ramos et al., 2018). Altitude hypoxia, overcoming normoxia and normobaric hypoxia, improved the velocity of a loaded movement, possibly due to the reduced density of air (Feriche et al., 2014).

The possible beneficial effects on power refer to acute exposure to moderate altitudes, as more severe acute hypoxia or long‐term hypoxia has been associated with muscle deterioration, reduced muscle function and loss of lean body mass (Feriche et al., 2014). Indeed, high‐altitude exposure has been reported to decrease muscle mass despite a sufficient intake of proteins (Bondi et al., 2021).

Hypoxia is included in those cumulative environmental exposures and associated biological responses that can affect the skeletal muscle system (Purcaro et al., 2024). From a neuromuscular perspective, conflicting results exist on hypoxia‐induced changes in muscle activation assessed via surface electromyography (Jordan et al., 2002; Nell et al., 2020; Rua et al., 2023; Saugy et al., 2016). Beyond individual muscle behavior, intermuscular coordination is affected by hypoxia: increased co‐contraction indices were observed after 48 h at 3600 m of altitude, interpreted either as a compensatory response of the nervous system to mitigate the loss of joint stability or due to a reduction in the efficiency of corticospinal control (Guerrero‐Henriquez et al., 2025). Moderate hypoxia elicited notable reduction in resting motor threshold, putatively due to membrane hyperexcitability of the cortical neurons (Márquez et al., 2023). Normobaric hypoxia at simulated moderate altitude stimulated the neuromuscular system, particularly by increasing motor units' discharge during maximal isometric contractions (Bondi et al., 2025).

Previous studies reported that standing balance is impaired consistently under hypobaric hypoxia, indicating impaired neuromuscular coordination attributed to disturbances in the central nervous system, affecting sensory integration from visual, vestibular, and somatosensory systems necessary for postural control (Wagner et al., 2011). Impairments are evident when visual cues are present, and this has been suggested to rely on the impaired ability of the central nervous system to maintain upright postural control by integrating and processing sensorimotor signals, exacerbated by parallel cognitive‐motor tasks (Debenham et al., 2021). However, impairments can be considered reversible, as a study reported overimposable results in static stabilometry after return to a high‐altitude expedition (Scordella et al., 2016).

In addition to postural control, other domains of neuromuscular coordination are supposed to be affected at high altitude, but literature is scarce on this topic. Running mechanics were only partially affected during a mountain ultramarathon due to hypoxia (Jeker et al., 2020), and not affected by altitude training at moderate altitude (Millet et al., 2021). For what concerns gait analysis, slower economical speed due to leftward shift of the U‐shaped relationship between oxygen cost per distance and speed was observed under severe normobaric hypoxia (Horiuchi et al., 2016).

Given this background, the aim of this project was to assess the response to high‐altitude hypoxia in terms of motor skills, by using static balance tests, jumping tests, gait analysis, and the measurement of maximal strength symmetry through portable technologies. In particular, given the continuous increase in high‐altitude tourism and the potential for exposure to high‐altitude hypoxia to induce neuromuscular alterations, this study aims to analyze, through a multimodal approach, different domains of functional motor skills in response to trekking up to 5000 m.

## 2 METHODS

### 2.1 Design and participants

**FIGURE 1:**
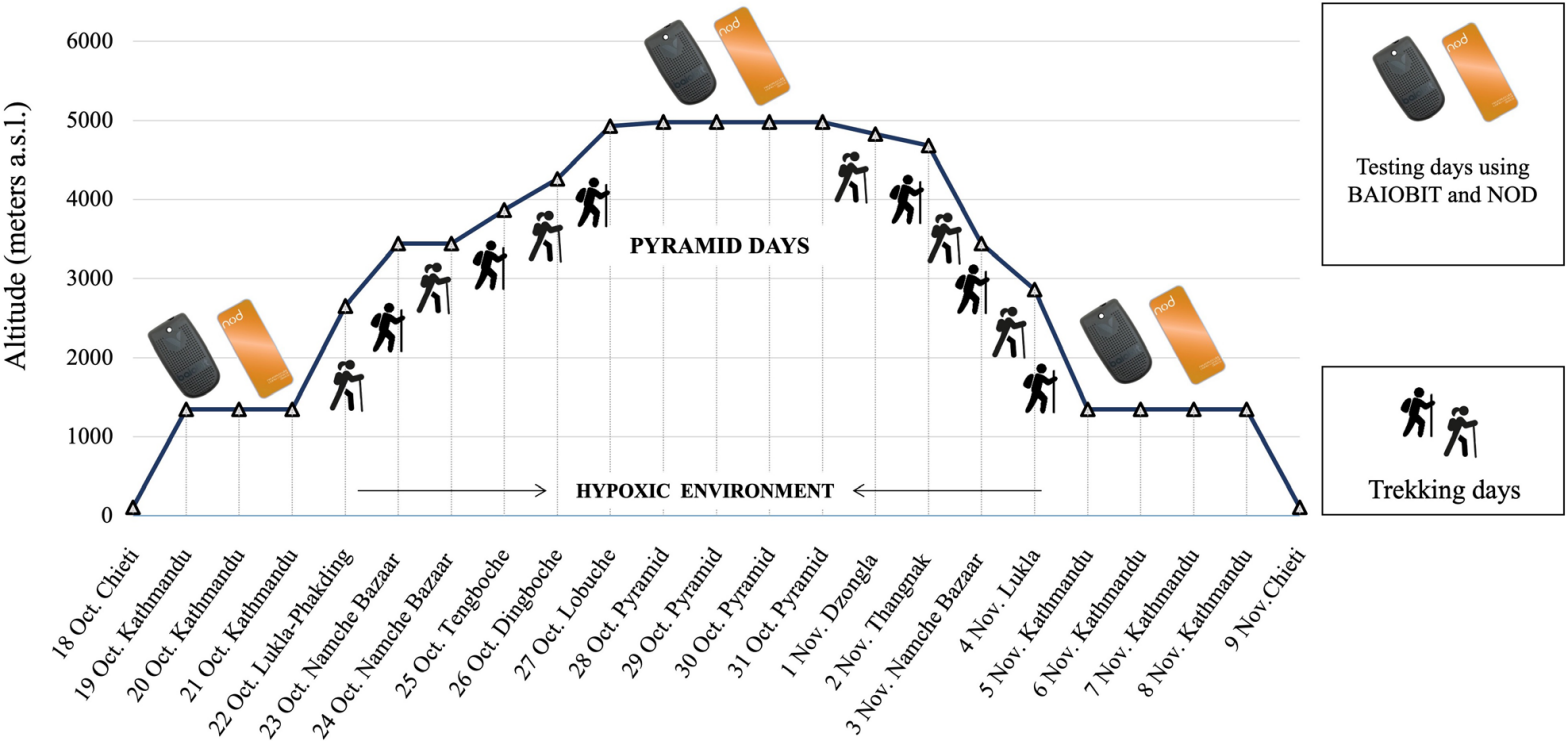
Study design and altitude program.

The low altitude tests were conducted in the Omkaar Polyclinic (Kathmandu, approximate altitude of 1400 m), both before (LA‐pre) and after (LA‐post) the high‐altitude trekking; the high‐altitude tests (HA) were conducted in the International Pyramid Laboratory (approximately altitude of 5000 m) (Figure 1). The ascent to the Pyramid Laboratory, commencing from Lukla Airport, was completed within a period of 6 days, with a designated rest day allocated in Namche Bazaar. The descent from Pyramid, following a five‐day sojourn, was completed within a span of 4 days. The route featured alternations between ascents and descents, with no utilization of ice axes or crampons. The backpacks carried by the participants generally exhibited a light weight, ranging from 3 to 10 kilograms. The whole project involved the 21 participants in the expedition; of these, 15 were chosen for the work in question due to logistical requirements and the possibility of completing the tests without hindrance. The sample consisted of healthy Italian travelers, 8 of whom were male and 7 female, with an age of 41.67 ± 14.71 years (range 23–67 years) and a body mass index of 23.91 ± 3.46 kg/m^2^ (range 18.78–28.96 kg/m^2^). All the participants successfully completed the expedition and took 250 mg of acetazolamide once daily during the ascent phase. None of them had diagnosis of active ischaemic heart disease, previous acute myocardial infarction, chronic obstructive pulmonary disease, psychosis, neurosis, schizophrenia, depression, alcoholism, drug addiction, neurological diseases, respiratory failure.

### 2.2 Instruments

The BAIOBIT device (Rivelo, BTS Bioengineering Group, Italy) includes a triaxial accelerometer (frequency bandwidth 4–1000 Hz) and gyroscope (bandwidth 4–8000 Hz), a magnetometer (bandwidth up to 100 Hz), and sensor fusion (bandwidth up to 200 Hz); inter‐instrument correlation coefficient ranges 0.90–0.99, has an intra‐instrument CoV ≤2.5%, and validity was demonstrated during double‐leg countermovement jumps (CMJs) (Camuncoli et al., 2022). It was used to test single‐leg balance with eyes open, walking at preferred speed, and repeated counter‐movement jumps. For all three tests, the device was positioned at the lumbar level using an adjustable strap. All three tests included a calibration phase for each trial.

NOD (OT Bioelettronica, Italy) is a multi‐purpose hand‐held device that can be used as a hand‐held dynamometer to measure the isometric strength of any muscles of the body districts (Boccia et al., 2017). Customized pads are to be applied over a thin rigid device with a parallelepiped shape, and the force is transmitted via wireless connection to a smartphone equipped with the dedicated app. It has been reported as a good surrogate to other dynamometers due to its portability, user‐friendly pairing to a smartphone app, and high inter‐reliability (Sartorio et al., 2025). NOD was used to test the symmetry of maximal isometric strength.

### 2.3 Procedures

All the tests were conducted in the morning. Tests were done on the second morning at high altitude to avoid the effects of hiking. The tests were supervised by the same two operators at all three test times. For all the tests, each participant was verbally instructed on the motor task. Balance was evaluated through a single‐leg stance test with the dominant leg and eyes open, 1 single trial lasting 30 s; sagittal, frontal, and transversal range of motion of pelvis were registered. The confidence area of the ellipse, rather than being used as an outcome variable, was used as an inclusion criterion for subsequent data analysis; those who used balancing strategies with excessive displacement of the supporting foot produced ellipsoids that were too large. The use of the contralateral foot as an aid during the test was considered a criterion for canceling the test.

Gait analysis was carried out on a minimum of 8 steps with preferred walking speed. Spatiotemporal features (speed, step length, flight time), symmetry and stability of gait (harmonic ratio, walking ratio), and pelvis movements (sagittal, frontal, and transverse range of motion) were registered. Due to logistical problem, gait analysis was carried out only at LA‐pre and HA. Vertical jump was evaluated through the execution of 5 double‐leg CMJs with hands on hips. Mean height (H mean), maximal height (H max), power, force and stiffness were registered.

Force symmetry was assessed using a maximum isometric plantar flexion test of the ankle, with a maximum of three trials on each side interspersed with 2 min. The asymmetry index (ASI) was calculated as follows:

$$\mathrm{ASI}=100–\left(\frac{{\mathrm{Max}}_{\text{left}}}{{\mathrm{Max}}_{\text{right}}}\times 100\right)$$

### 2.4 Statistical analyses

The normality of the residuals was verified using the Kolmogorov–Smirnov test and by observing the Q–Q plots. After possible removal of outliers, repeated measures analyses were conducted by using either RM‐ANOVA or mixed‐effect analysis for jump and balance tests, with post‐hoc comparisons corrected for multiple comparisons with the Tukey method; for the walking test, comparisons were made using paired *t*‐tests; in all cases, the tests were performed after verifying normality. ASI Analyses and graphs were performed using Prism Version 10 (GraphPad Software, USA). In light of the ecological nature of the research, rather than a determination of the sample size, a sensitivity analysis was conducted to ascertain the required effect size by using software G*Power v. 3.1.9.3. For LA vs. HA comparisons, given *α* = 0.05, 1–*β* = 0.80, and 15 participants, the required effect size *d* is 0.778; for LA vs. HA vs. LA, given *α* = 0.05, 1–*β* = 0.80, and 15 participants, the required effect size *f* is 0.346.

## 3 RESULTS

**FIGURE 2:**
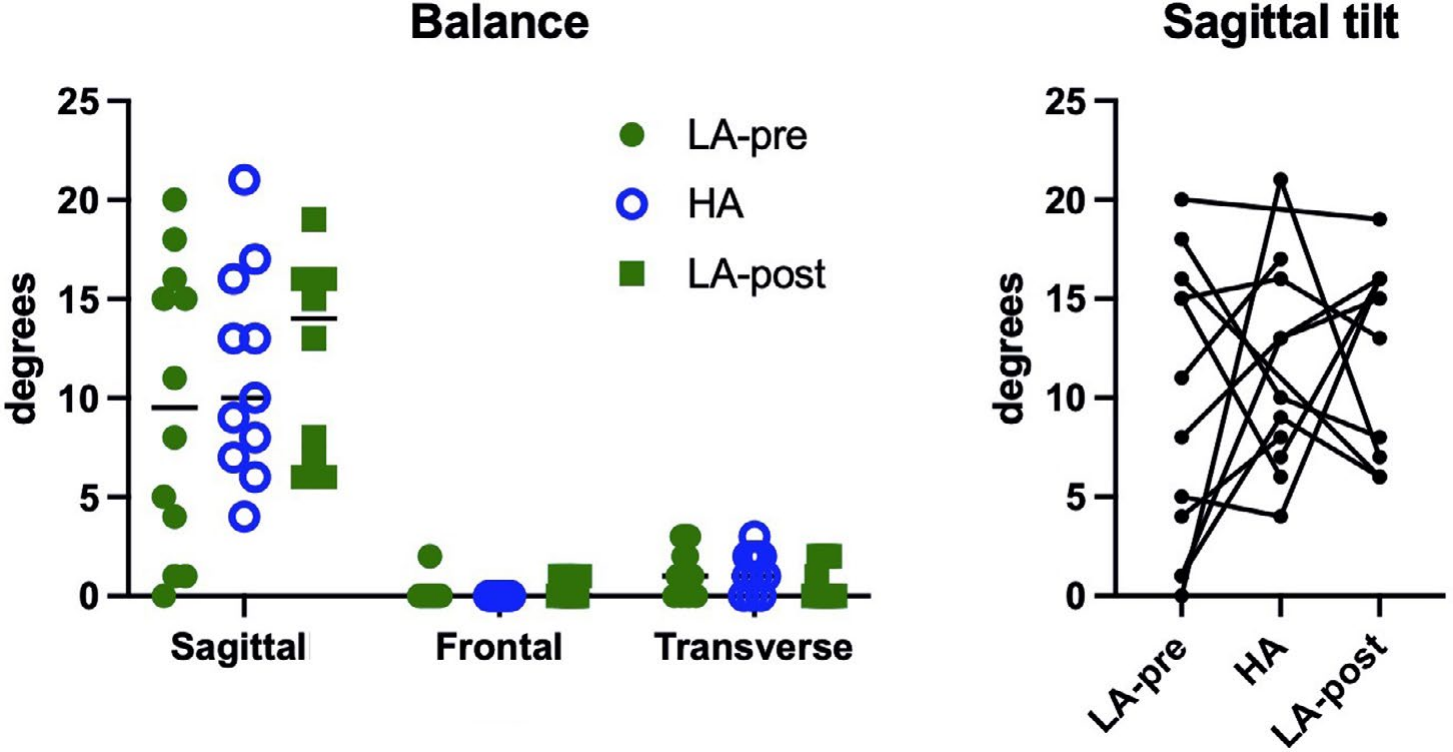
Results of 3d hip movements during the single leg static balance test across the three study times. The panel on the left shows the range of motion on the three planes during the three test moments; the panel on the right focuses on sagittal movements with the changes of each individual.

With regard to the single‐leg balance test, the posture maintenance strategy developed mainly with sagittal tilt, with heterogeneous changes among participants between the three times (*p* = 0.554, *f* < 0.001) (Figure 2).

**FIGURE 3:**
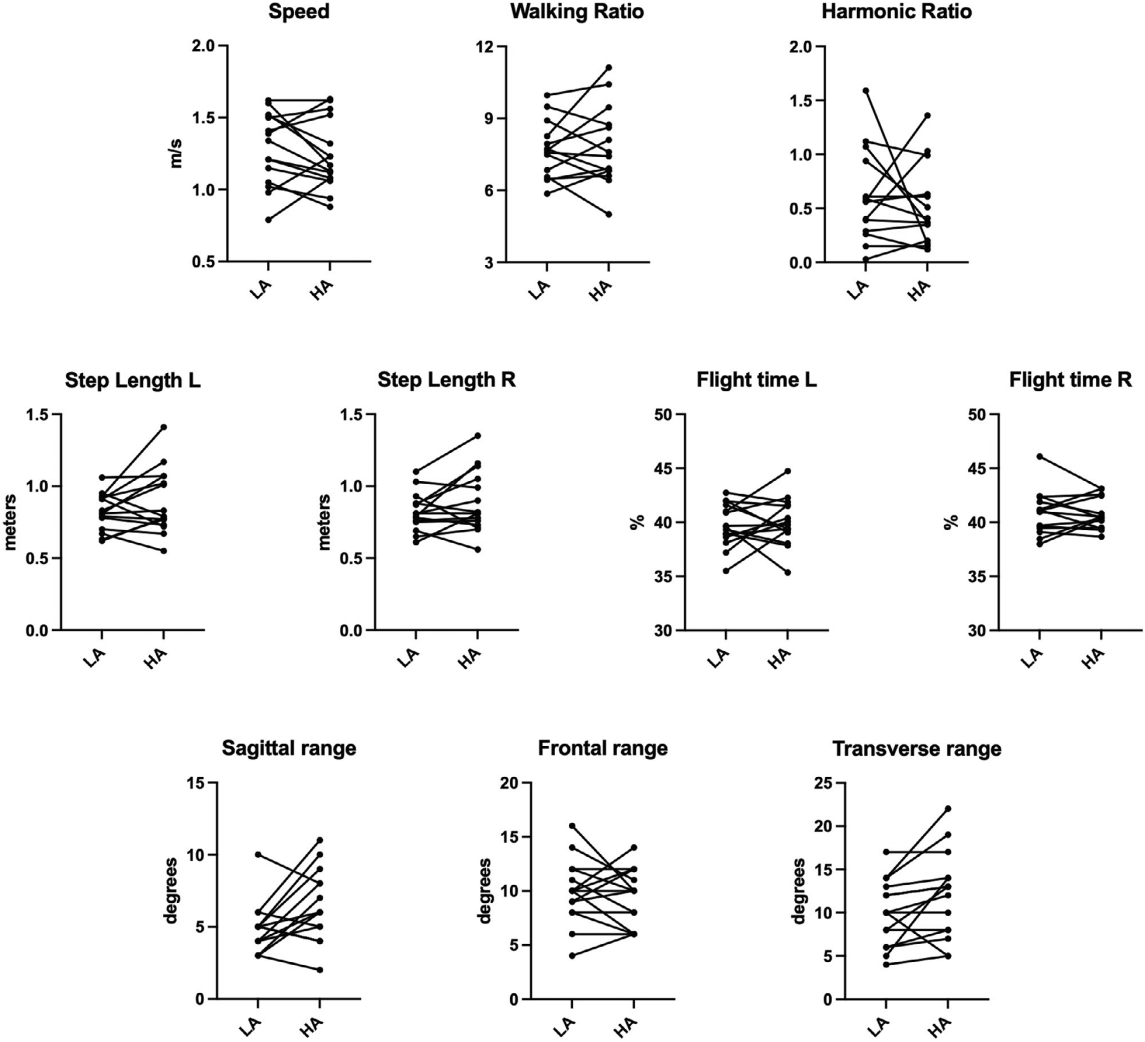
Results of gait analysis from the lumbar IMU across the three study times. A line was traced for each participant to illustrate the individual variations between the two test times.

In the walking test (Figure 3), mean preferred walking speed was 1.29 ± 0.25 m/s at LA‐pre and 1.24 ± 0.24 m/s. There were no appreciable changes between low and high altitude for Walking Ratio (*p* = 0.557, Cohen's *d* = 0.161), Harmonic Ratio (*p* = 0.526, *d* = 0.174), speed (*p* = 0.379, *d* = 0.235), stride length (left: *p* = 0.162, *d* = 0.382; right: *p* = 0.161, *d* = 0.383) and flight time (left: *p* = 0.649, *d* = 0.120; right: *p* = 0.924, *d* = 0.026); the RoM at the hip level varied significantly, with average increases both in the sagittal plane (*p* = 0.042, *d* = 0.579, although a reduction was found in one third of participants) and in the transverse plane (*p* = 0.037, *d* = 0.595), while in the frontal plane the response was heterogeneous among participants (*p* = 0.469, *d* = 0.192). None of the comparisons exceeded the minimum required effect size.

**FIGURE 4:**
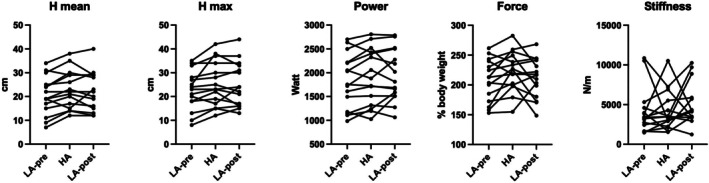
Results of vertical jump across the three study times. A line was traced for each participant to illustrate the individual variations between the three test times.

With regard to vertical jump (Figure 4), there was a progressive increase from LA‐pre to HA, with maintenance at LA‐post in both average height (from 20.40 ± 7.94 to 23.00 ± 8.00 and 22.47 ± 8.23 cm, *p* < 0.001, *f* = 0.576) and maximum height (from 22.47 ± 8.30 to 25.33 ± 9.26 and 25.13 ± 9.29 cm, *p* = 0.007, *f* = 0.573), accompanied by statistically non‐significant increases in power (*p* = 0.086, *f* = 0.341), while changes in stiffness were not consistent across subjects (*p* = 0.263, *f* = 0.165). Both the comparison for average height and maximum height exceeded the minimum effect size required. Average height increased from LA‐pre to HA on average of 2.60 cm (95% CI 1.02–4.18, *p*_Tukey_ = 0.009) and remained stable once back in Kathmandu (*p*_Tukey_ = 0.820). Similarly, maximal height increased from LA‐pre to HA on average of 2.87 cm (95% CI 0.82–4.92, p_Tukey_ = 0.024) and remained stable once back in Kathmandu (*p*_Tukey_ = 0.971).

**FIGURE 5:**
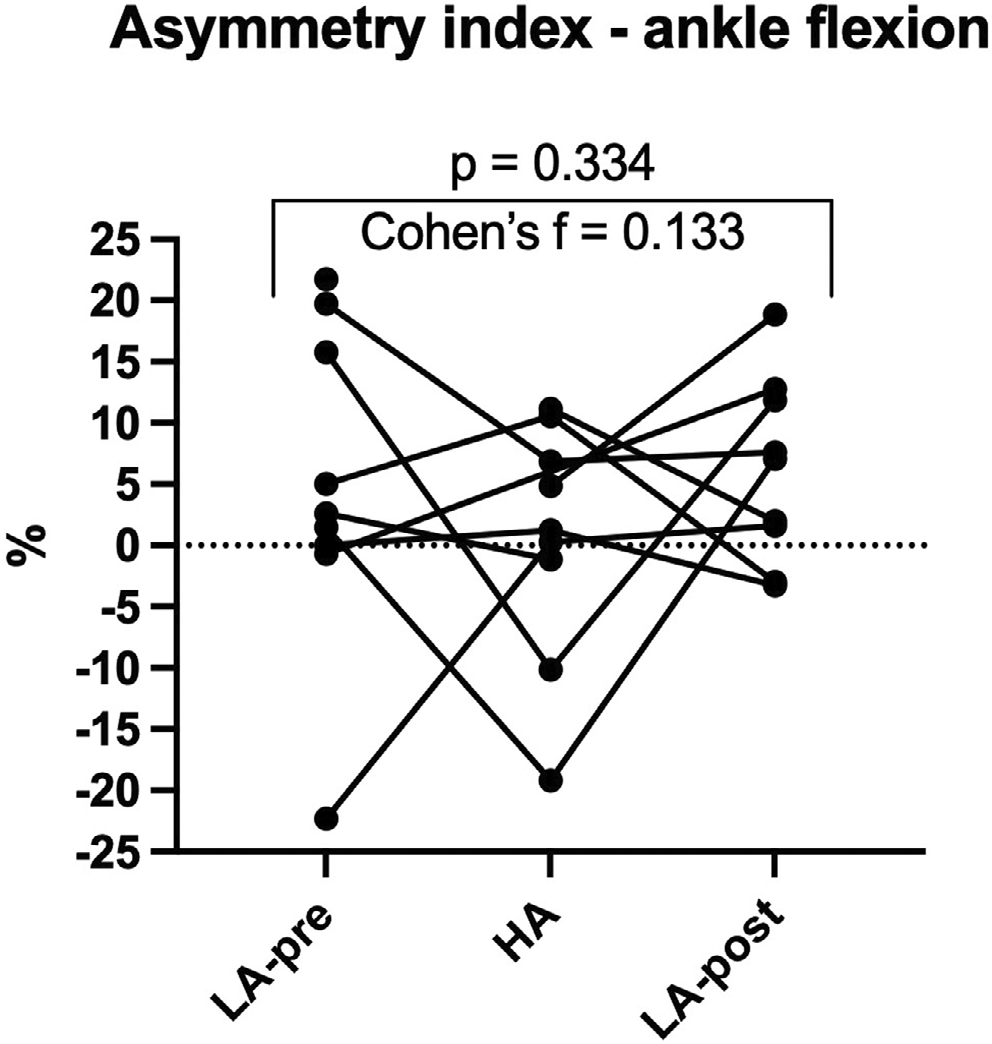
Results of inter‐limb asymmetry index calculated on maximal isometric strength of calf muscles across the three study times. A line was traced for each participant to illustrate the individual variations between the three test times.

No homogeneous trend across participants was found in the ASI (Figure 5). Differences among the two sides remained in a broad range, suggesting large inter‐individual heterogeneity in the strength asymmetry and in altitude trekking‐dependent changes.

## 4 DISCUSSION

This study contributes to the understanding of how days‐lasting trekking at high altitude with a light backpack influences gait dynamics and stability, vertical jumping performances, and triceps surii's maximal strength symmetry.

Gait stability was evaluated through walking ratio, which is based on spatiotemporal features as the ratio of step length (mm) to cadence (steps/min) and it falls usually in range 6–7 mm/steps per minutes in healthy young people (Sekiya & Nagasaki, 1998), and harmonic ratio, which is based on trunk accelerations in the frequency domain and it is also used to assess smoothness and step‐to‐step symmetry. Impairments in gait control likely lower harmonic ratio and walking ratio. On average, we did not observe such impairments across the study times. These results couple with those on single‐leg balance test and demonstrate that the multi‐day trekking at hypobaric hypoxia does not impair static and balance neuromuscular control, at least up to 5000 m. Notably, we observed increased hip movements in both the sagittal and transverse planes during walking among most participants, indicating a more flexible, yet adequately controlled, gait pattern in response to high‐altitude trekking while carrying a light backpack.

Beyond gait, enhanced performance in rapid actions is expected as altitude increases, mainly due to lower air density, although very high altitude impairs muscular performances (Feriche et al., 2014). However, the increments of vertical jump height due to the decrease of air density can be considered negligible, and the reduction of gravitational magnitude can be considered almost worthless (Bondi, 2023). In particular, the drag force opposing the body's movement through the air is lower during vertical jumps thanks to the body's shape, which is fairly streamlined. The results obtained in this study are interpreted as the effect of performance supercompensation in response to multi‐day trekking on predominantly uphill routes with very low overload.

For what concerns limb strength asymmetry, no specific threshold has been defined for judging normal asymmetry, despite usually a maximum of 10%–15% that can be considered a target for rehabilitation (Parkinson et al., 2021). The results obtained show a marked alteration in the asymmetry index between the study times, with a marked inter‐individual difference in the changes and a significant amount of asymmetry exceeding 15%. These trends are interpreted as the result of an expression of poorly stable plantar flexion strength, combined with the difficulty of standardization with field dynamometry.

The study came with inherent limitations, as its nature itself, i.e., a field study conducted in non‐standardized laboratories did not allow to set the test conditions adequately. The gait analysis was not conducted upon return to Kathmandu because the testing facilities did not have at least 10 linear metres of unobstructed space. The maximum strength test was evaluated solely in terms of symmetry, as the nature of the device (a hand‐held dynamometer adaptable to different muscle groups, with experimenter‐dependent detection) and the impossibility of having an identical context between tests would have led to biased comparisons. A further limitation of the study design is that it precludes the separation of the exercise‐to‐altitude effect; it is possible that even the learning effect from repeated testing may have occurred. The combination of this limitation with the absence of a control group hinders the ability to infer causality. The limited sample size may have obscured the presence of heterogeneity due to factors such as age, sex, or physical fitness, which cannot be statistically assessed. The utilization of acetazolamide is regarded as a confounder in physical performance evaluation, given its impact on acid–base equilibrium and respiration.

In conclusion, high‐altitude trek improved vertical jump, despite the possible impairment effect of days‐lasting hypoxia, putatively not due to reduced air density. Gait features and stability, as static balance and strength symmetry, varied across participants with no homogeneous trends across study times. The employment of appropriately validated portable instruments signifies a noteworthy advancement in the realm of motor control and physical performance research at high altitude. In future expeditions, portable instruments can be used to highlight any altitude thresholds at which motor deficits become more evident, thereby enabling the development of easily implementable tests to support evidence‐based individual modifications to altitude plans.

## AUTHOR CONTRIBUTIONS

**Michele D'Attilio:** Conceptualization; formal analysis; funding acquisition; investigation; methodology; resources. **Riccardo Rua:** Investigation; methodology. **Alberto Taverna:** Investigation. **Vittore Verratti:** Funding acquisition; project administration; resources; supervision. **Danilo Bondi:** Conceptualization; formal analysis; investigation; methodology; visualization.

## FUNDING INFORMATION

DB was supported by Vivisol S.r.l. (Monza, Italy). The funders had no role in the design of the study, data collection, analyses, or interpretation of the data, writing of the manuscript, or in the decision to publish the results.

## CONFLICT OF INTEREST STATEMENT

The authors declare no conflict of interest.

## ETHICS STATEMENT

All study procedures were conducted in accordance with the 1964 Declaration of Helsinki and its subsequent amendments. This work is an ancillary part of a study approved by the local Institutional Review Board (‘Comitato Etico delle Province di Chieti e Pescara’, document no. 18, 29/07/2021). Written informed consent was obtained from each study participant.

## Data Availability

Data will be available upon reasonable request to the corresponding author.
